# Providing high-quality care remotely to patients with rare bone diseases during COVID-19 pandemic

**DOI:** 10.1186/s13023-020-01513-6

**Published:** 2020-08-31

**Authors:** E. Brizola, G. Adami, G. I. Baroncelli, M. F. Bedeschi, P. Berardi, S. Boero, M. L. Brandi, L. Casareto, E. Castagnola, P. Fraschini, D. Gatti, S. Giannini, M. V. Gonfiantini, V. Landoni, A. Magrelli, G. Mantovani, M. B. Michelis, L. A. Nasto, L. Panzeri, E. Pianigiani, A. Scopinaro, L. Trespidi, A. Vianello, G. Zampino, L. Sangiorgi

**Affiliations:** 1 Department of Rare Skeletal Disorders, IRCCS Istituto Ortopedico Rizzoli, Bologna, Italy; 2 Rheumatology Unit, University of Verona, Verona, Italy; 3 Division of Pediatrics, Department of Obstetrics, Gynecology and Pediatrics, University Hospital, Pisa, Italy; 4 Medical Genetic Unit, Fondazione IRCCS Ca’ Granda, Ospedale Maggiore Policlinico, Milan, Italy; 5 Italian Osteogenesis Imperfecta Association (As.It.O.I), Olgiate Molgora (LC), Italy; 6 Department of Paediatric Orthopaedics, IRCCS Giannina Gaslini Institute, Children’s Hospital, Genoa, Italy; 7 Department of Experimental Biochemical and Clinical Sciences, University of Florence and Fondazione FIRMO, Florence, Italy; 8 Infectious Disease Department, Giannina Gaslini Institute, IRCCS, Genoa, Italy; 9 Scientific Institute, IRCCS E. Medea, Bosisio Parini (LC), Italy; 10 Department of Medicine, Clinica Medica 1, University of Padova and Regional Centre for Osteoporosis, Padua, Italy; 11 Rare Diseases and Medical Genetics Unit, Bambino Gesù Children’s Hospital, IRCCS, Rome, Italy; 12 Valduce Hospital - Villa Beretta Rehabilitation Centre, Lecco, Italy; 13 National Center for Drug, Research and Evaluation, Istituto Superiore di Sanità, Rome, Italy; 14 Endocrinology Unit, Fondazione IRCCS Ca’ Granda Ospedale Maggiore Policlinico, Milan, Italy; 15 Department of Clinical Sciences and Community Health, University of Milan, Milan, Italy; 16 Italian Federation of Rare Diseases Patients Associations (UNIAMO FIMR), Rome, Italy; 17 Obstetrics and Gynecologic Unit, Fondazione IRCCS Ca’ Granda, Ospedale Maggiore Policlinico, Milan, Italy; 18 Respiratory Pathophysiology Division, University of Padua, Padua, Italy; 19 Rare Diseases and Birth Defects Unit, Dipartimento di Scienza della Salute della Donna, del Bambino e di Sanità Pubblica, Fondazione Policlinico Universitario A. Gemelli IRCCS, Università Cattolica del Sacro Cuore, Rome, Italy; 20 Department of Rare Skeletal Disorders & CLIBI Laboratory, IRCCS Istituto Ortopedico Rizzoli, Bologna, Italy

**Keywords:** 2019-nCoV, Bone diseases, Care, Coronavirus, COVID-19, ERN, Rare diseases, Remote

## Abstract

During the COVID-19 outbreak, the European Reference Network on Rare Bone Diseases (ERN BOND) coordination team and Italian rare bone diseases healthcare professionals created the “COVID-19 Helpline for Rare Bone Diseases” in an attempt to provide high-quality information and expertise on rare bone diseases remotely to patients and healthcare professionals. The present position statement describes the key characteristics of the Helpline initiative, along with the main aspects and topics that recurrently emerged as central for rare bone diseases patients and professionals. The main topics highlighted are general recommendations, pulmonary complications, drug treatment, trauma, pregnancy, children and elderly people, and patient associations role. The successful experience of the “COVID-19 Helpline for Rare Bone Diseases” launched in Italy could serve as a primer of gold-standard remote care for rare bone diseases for the other European countries and globally. Furthermore, similar COVID-19 helplines could be considered and applied for other rare diseases in order to implement remote patients’ care.

## Background

The coronavirus disease 2019 (COVID-19) caused by the novel coronavirus SARS-CoV-2 had its outbreak in December 2019 in China. As per the World Health Organization (WHO) situation report of April 18th 2020, the outbreak stands at more than 2.1 million infected people leading to more than 145.000 deaths globally, and it is rapidly increasing [1]. Common symptoms of COVID-19 disease include fever, dry cough, dyspnoea, vomiting, diarrhoea, fatigue and myalgia [2, 3].

Symptoms can take from 2 to 14 days to appear after exposure. The virus is spread by respiratory droplets (generated from coughs or sneezes) through close contact with an infected person or touching surfaces exposed to the virus. The majority of the cases (> 80%) are asymptomatic or have a relatively mild course being self-managed at home. However, around 20% undergo more severe and complicated outcomes once the virus affects the lower respiratory tract and manifests as severe pneumonia [3, 4]. Up to now, in Europe there were more than 1.086.000 positive confirmed cases and at least 65% of the global deaths occurred in European countries, with Italy registering the highest death toll in EU [1].

In Europe, a disease is defined as rare when it affects no more than 5 in 10.000 people [5] and it is estimated that 6–8% of the population has a rare disease. Patients with rare bone diseases (RBD) need continuous high quality care and careful multidisciplinary follow-up as several comorbidities and complications may be associated to the primary musculoskeletal abnormality, affecting other body systems such as the pulmonary, cardiac, vascular, neurologic system. During the COVID-19 pandemic, unprecedent public health measures were taken in order to restrain the outbreak, such as the lockdown of several cities/countries and the self-isolation of individuals in their home [6, 7].

Once these difficult but necessary decisions were taken, the impact on healthcare systems was immediate and a rearrangement of structure and priorities was put into practice. Most of the healthcare workers were moved from their departments to the front line of the new COVID-19 units, creating a gap in many other medical sectors, and thus many medical visits and pre-scheduled exams and surgeries were canceled or postponed. Many patients with RBD experienced a feeling of fear, abandon and faced discontinuation of treatment. On the other hand, many healthcare professionals with scarce or no experience with RBD, had to provide immediate care or apply preventive measures for RBD patients. In order to tackle this emergency, the European Reference Network on Rare Bone Diseases (ERN BOND) coordination team and Italian RBD healthcare professionals created the “COVID-19 Helpline for Rare Bone Diseases” in an attempt to provide high quality-information and expertise on RBD remotely to patients and healthcare professionals.

## Helpline initiative - overview

ERN BOND brings together 78 experts on RBD representing 38 specialized centers and 4 European Patient Advocacy Groups (ePAGs) from 10 European states. The main goal is to ensure the best patient-centered multidisciplinary care and the gold-standard of care to individuals with RBD [8].

The ERN BOND coordination team and Italian RBD healthcare professionals set up a dedicated 24/7 telephone line the “COVID-19 Helpline for Rare Bone Diseases”. The Helpline provides high-quality information and expertise on RBD remotely to patients and healthcare professionals, with particular reference to those working in intensive care units and / or COVID-19 units. Although we have received many calls from healthcare professionals (around 20%), the majority of the calls were made by patients or patients’ family members. Initially, the COVID-19 Helpline was created to provide information only in Italian language, however, we also received and responded to calls from healthcare professionals in English language.

The Helpline was extremely successful and over 200 telephone calls and messages were received. We received around 10 calls/day, however, to date, we have seen a higher number of calls at two specific times: when the lockdown officially started and when there was a peak in COVID-19 positive cases. Some requests were unique, however different key-aspects and questions recurred frequently and were indeed prevalent. Healthcare professionals’ main requests include invasive procedures and abnormal anatomy due to bone deformities. While patients’ requests were more related to drug treatment (schedule, prescription, etc.) or to find local physicians to be able to keep a continued follow-up in cases of pain, fractures or joint subluxation.

The Italian RBD experts decided to create this position statement to summarize and share the key aspects and recommendations that stood out through this important initiative based on a combination of the available evidence, good practice and expert advice.

## Helpline characteristics

In order to provide a remote high standard of care, some specific professional and technical characteristic are required. First, the professionals that answered the calls and handled the request were all healthcare professionals (physicians, nurses, physiotherapist, etc.) with at least 5 years of experience in RBD. Shifts in order to provide 1 professional available for calls were organized. Second, even though social media and chats are more and more common in remote care, the decision of using telephone allowed all patients to communicate directly, rapidly and without any digital divide (in particular for elderly patients). Third, the service was entirely free of charge, allowing access to the service to all individuals from any socio-economic conditions. Fourth, the service was provided 24/7 as emergency situations such as the COVID-19 pandemic require immediate attention and the calls cannot be pre-scheduled. Fifth, the service was provided in the local language (Italian), in order to avoid any language barriers.

## RBD & COVID-19: key-aspects & recommendations

### General recommendations

The general recommendations from WHO are applicable to all individuals including those with RBD [7]. The main recommendations are:

- Wash the hands frequently
- Maintain social distancing
- Avoid touching eyes, nose and mouth
- Practice respiratory hygiene (it means covering mouth and nose with the bent elbow or a tissue when coughing or sneezing)
- Stay informed and follow the advice given by the healthcare provider
- In case of fever, cough and difficult breathing, seek medical care early

### Pulmonary complications

Critically ill patients with SARS-CoV-2 pneumonia may develop Acute Respiratory Distress Syndrome (ARDS) and require supportive treatment [9]. Current supportive treatment includes the following:

- High-Flow Nasal Cannula (HFNC) oxygen therapy: HFNC delivers heated, humidified inspired gas at a high flow rate and a precise fraction of inspired oxygen (FiO_2_). The device is being increasingly utilized to correct severe, refractory hypoxemia in patients with respiratory distress due to a variety of causes [10]. Although there is widespread concern about a possible increased risk of viral transmission [11], recent guidelines suggest utilization of HFNC oxygen therapy to treat critically ill subjects with SARS-CoV-2 pneumonia who are not responding to conventional oxygen therapy [12].
- Non-Invasive Ventilation (NIV): the application of NIV in patients with SARS-CoV-2 pneumonia is controversial. The non-invasive ventilatory approach is currently not recommended for patients with viral infections complicated by pneumonia because NIV does not necessarily change the natural disease course [13]; on the other hand, NIV has been shown to be effective in the management of Acute Respiratory Failure (ARF) in subjects with Osteogenesis Imperfecta (OI) [14]. A trial of NIV has been suggested in patients with SARS-CoV-2 pneumonia if HFNC is not available [12]; full face or total face mask should be preferred for connecting a patient to the ventilator. Close monitoring and short-interval assessment for worsening of respiratory failure are recommended [12].
- Endotracheal intubation (ETI) and Invasive Mechanical Ventilation (IMV): ETI may be particularly difficult and unsafe in individuals with OI and should be performed by a trained and experienced professional, as these patients are known to have difficult airway with a high risk of odonto-axial dislocation and cervical, mandibular and tooth fractures during direct laryngoscopy [15]. Patients with severe pneumonia may desaturate quickly during intubation and should be pre-oxygenated with 100% FiO_2_ for 5 min, via a face mask with reservoir bag, bag-valve mask, HFNC or NIV. Video guided laryngoscopy is suggested, if available, to reduce the risk of infection for providers [12].
- In case of difficult airways, the use of fiberoptic bronchoscopy is suggested to facilitate ETI. In mechanically ventilated adults with COVID-19 and ARDS, utilization of low tidal volume (Vt) ventilation (Vt 4–8 mL/kg of predicted body weight) is recommended, combined with high Positive End-Expiratory Pressure strategy [12].

**Fig. 1:**
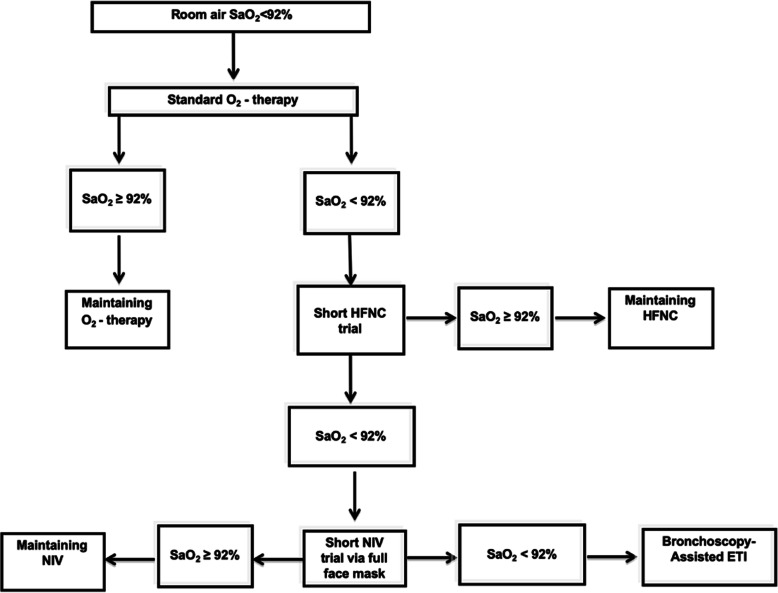
Treatment algorithm to reverse hypoxemia in OI patients with severe ARF associated to SARS-CoV-2 pneumonia. ETI = endotracheal intubation; HFNC = high-flow nasal cannula; NIV = non-invasive ventilation; SaO_2_ = arterial oxygen saturation

A treatment algorithm based on a stepwise utilization of HFNC, NIV and ETI can be utilized to reverse hypoxemia in patients with OI who develop severe ARF associated to SARS-CoV-2 pneumonia (Fig. 1).

### Drug treatments

Access to therapies and specialists’ consultation has become challenging during the COVID-19 pandemic, with even more relevant problems for patients affected by rare disorders. Indeed, this fragile population is, by definition, more brittle and tend to suffer more from any additional health problem. Patients can “forget” their disease, being, instead, concentrated on avoiding the risk of infection. Moreover, patients might incautiously discontinue their treatments, especially if the disease is not immediately life threatening. For this reason, it is necessary to offer specific indications related to the treatments to RBD patients during this COVID-19 outbreak.

First, patients taking vitamin D and calcium, should continue their treatment, especially considering the postulated extra-skeletal effects of vitamin D on innate and adaptive immunological response. Indeed, vitamin D supplementation could protect against acute respiratory tract infection [16] and, even more interestingly for COVID-19, has been shown to reduce circulating IL-6 in critically ill patients [17]. In addition, quarantined individuals are usually less exposed to sunlight and, in turn, the synthesis of vitamin D is diminished [18]. Therefore, we suggest to adults to take more than the usually recommended 1000 IU of daily cholecalciferol [19] and to paediatricians to adjust the dosage in children as well [20]. With regards to calcium intake, we suggest to follow current recommendations on age-related calcium intake.

Children with vitamin D-dependent rickets type 1 and type 2 should continue the regimen of treatment with active vitamin D metabolites, such as calcitriol or alfacalcidol, in combination with calcium salts [21]. Furthermore, children with X-linked hypophosphatemic rickets receiving conventional treatment (calcitriol or alfacalcidol associated with inorganic oral phosphate salts) [21] or the recent new Burosumab therapy [22–24] must be accurately followed by the bone specialist performing the scheduled checks.

Second, we recommend to patients on amino bisphosphonates (i.e., OI patients both adults and children) [25, 26] not to discontinue their treatment. Intravenous amino bisphosphonates infusions can be safely postponed, or, in exceptional situations, an oral substitutive agent can be offered to those that cannot delay the treatment. Nevertheless, bone specialists should be consulted through telehealth service and a shared decision-making strategy should be encouraged.

Finally, for patients under treatment with orphan drugs the therapy should be continued independently of the actual crisis with the agreement of the bone specialist.

### Trauma

In order to create COVID-19 emergency units, hospital settings were re-organized to free up resources (i.e. ICU beds, blood products, ventilators, personal protective equipment) and all elective orthopaedic procedures have been cancelled or postponed, with the only exception of tumour surgery.

Minor injuries (i.e. contusions, ankle sprains, simple wounds) should be treated by an Accident and Emergency (A&E) consultant without input of an orthopaedic surgeon. As well as, the cases that do not need treatment or need a simple bandage or splint application. Imaging exams should be performed in the A&E department and, if needed, the orthopaedic surgeon should review it remotely. If an orthopaedic consultation is necessary, it should be performed in the A&E department by the orthopaedic surgeon wearing full personal protective equipment (PPE) [27]. Procedures like conscious sedations for fracture reduction or reduction of dislocations should also be performed in the A&E department without transferring the patient to other areas of the hospital. Before discharge from the A&E department “at-risk” patients and their accompanying parent (if the patient is a child) should be offered a swab test for SARS-CoV-2 infection. Patients should be instructed to go home and respect a quarantine period until the result of the swab test is available. Contact number and address of the patient is forwarded to the local health authority for further follow-up in the community.

Major injuries or emergency cases requiring surgical treatment should be directly transferred from A&E to SARS-CoV-2 dedicated operating room through a dedicated entrance and elevator. Following surgery, patients are transferred to a dedicated multidisciplinary COVID-19 ward.

A pre-operative swab for SARS-CoV-2 should be performed in all “at-risk” trauma patients before surgery and every effort should be made to postpone surgery until the result of the swab is available. Thus, if the result is negative, the patient can be treated and transferred to the regular orthopaedic ward, and if it is positive, surgery is performed speedly and post-operative care is provided in the dedicated COVID-19 ward.

The adoption of the above mentioned guidelines has been recommended to all the orthopaedic units [27, 28] and has been successfully in place for the last 4 weeks at the Giannina Gaslini Children’s Hospital (Genoa, Italy) providing 24/7 coverage of all paediatric (< 14 years-old) trauma cases in the northern region and elective care treatment of all ailments of the musculoskeletal system below the age of 18 (including upper limb and lower limb reconstructive, tumour, and spinal surgery).

### Pregnancy

Pregnancy in woman with RBD may increase the risk of complications associated with the condition, such as cardiopulmonary and musculoskeletal problems. Thus, pregnant RBD patients have a high-risk pregnancy which requires a multidisciplinary follow-up [29–31].

To date, there is no scientific evidence about an increased susceptibility of pregnant women to COVID-19. However, there is epidemiologic evidence that a small portion of pregnant women is at higher risk of severe illness from viral infections [32].

Pregnancy is a physiological state that predisposes women to respiratory complications of viral infection. Due to the physiological changes in their immune and cardiopulmonary systems, pregnant women are more likely to develop severe illness after infection with respiratory viruses [33]. Additionally, the presence of a pre-existing (or diagnosed during pregnancy) heart disease or respiratory problem, might increase the development of complications from COVID-19. Risk estimation for cardiovascular disease, in general, is mostly based on functional cardiopulmonary status. It was evident that pregnant women with other viral pneumoniae had an increased risk of preterm birth, fetal growth restriction and having a newborn with low birth weight [33].

Currently, there is no evidence that SARS-CoV-2 can be transmitted to the unborn child during pregnancy, or during childbirth (vertical transmission) [34–36].

In light of the above, all pregnant women should be extra careful to avoid infection. Preventive actions include social distancing measures and washing hands often using soap and water or alcohol-based hand sanitizer [27]. Stringent measures are required for women with a severe cardiorespiratory complication secondary to RBD.

If a pregnant woman or anyone in her household has symptoms of cough, fever and difficult breathing, or has been diagnosed with COVID-19, it is necessary to contact the maternity service to organize the right place and time for the visits.

It should be kept in mind that maternity care is an essential service and should be planned along with other essential services. The risks of not attending care include harm to women, their baby or both of them, even in the context of COVID-19 global pandemic. Suspected, probable and confirmed cases of SARS-CoV-2 infection should be treated in hospitals with effective isolation facilities and protection equipment. Suspected/probable cases should be treated in isolation and confirmed cases should be managed in a negative-pressure isolation room. They are called negative pressure rooms because the air pressure inside the room is lower than the air pressure outside the room. This means that when the door is open, potentially contaminated air or other dangerous particles from inside the room will not flow out into the uncontaminated areas. A critically ill confirmed case should be admitted to a negative-pressure isolation room in the intensive care unit.

It is important that pregnant women with RBD continue to attend their scheduled routine care and follow-up for assessment for complications RBD-related. Management of SARS-CoV-2 infected pregnant women should be undertaken by a multidisciplinary team (obstetricians, maternal– fetal-medicine subspecialists, obstetric anaesthetists, midwives, virologists, microbiologists, neonatologists, infectious-disease specialists).

At delivery if the mother has COVID-19, the newborn should be test with pharyngeal swab.

For the newborn with skeletal disease with involvement of thorax and a potential reduction of compliance of lung, temporary separation of the newborn from a mother could be useful to reduce the risk of mother-baby transmission, until the result of neonatal swab.

If the swab is positive for coronavirus separation is not long necessary and the neonate can stay with mother. If the swab is negative, the risk and benefits of prolonging separation should be discussed with the mother by clinicians and decision should be made in accordance with mother’ desires. If separation is not accepted, the mother should take all possible precautions to avoid spreading the virus to her infant, including her hands before touching the infant and wearing a face mask while feeding at the breast.

We do not know whether mothers with COVID-19 can transmit the virus via breast milk, however the virus has been detected in the breast milk of woman with COVID-19 [37].

Whether and how to start or continue breastfeeding should be determined by the mother in coordination with her family and healthcare providers.

### Children and elderly people

Children and adolescents are vulnerable to SARS-CoV-2 infection, but the disease appears to be less severe than in adults [38, 39]. The reason for this different severity remains to be determined. Differences in the clinical presentation of COVID-19 in children and adults have been reported [38, 40, 41]. Prevalence of clinical features among children and adults with COVID-19 is listed in Table 1.

**Table 1:** Comparison of prevalence of clinical features in children and adults with COVID-19

Symptoms	Children, % *n* = 36	Adults, % *n* = 175
Fever	36	86
Cough	19	62
Pharyngeal congestion	3	5
Dyspnoea	3	13
Pneumonia	53	95
Leucopenia	19	25
Lymphopenia	31	35
Increased myocardial enzymes	31	22
Increased liver enzymes	6	18
Increased C-reactive protein	3	49
Asymptomatic	28	< 5

Data are presented as percentage (%). Note: modified from Qiu et al., 2020 ^40^

Common abnormal laboratory findings that have been described in SARS-COV-2 infected patients are elevated creatine kinase MB, lymphopenia and leucopenia. Comparing children and adults, an elevated procalcitonin is described in pediatric patients and not common in adults, whereas adults present more often an increase in C-reactive protein than children. In pediatric patients, variables that are associated with severity of COVID-19 are radiographic presentation, decreased lymphocytes count, elevated body temperature, and high levels of procalcitonin, D-dimer, and creatine kinase MB [38, 41]. Most common radiologic findings are bilateral ground-glass opacities. Consolidation with surrounding halo sign may be considered as a typical sign in pediatric patients [38].

Large Chinese pediatric case series have documented only two cases of death in children [42, 43], but in the last days, mass-media reported the death of some children and adolescents both in European countries and in the United States. Children may be a disease spreader for more vulnerable people, such as grandparents, therefore having a critical role in community-based viral transmission, even without showing symptoms [44, 45].

Reports about SARS-CoV-2 infection in children and adolescents with chronic diseases or RBD, are actually lacking. They may likely be infected in the same proportion as healthy children and adolescents. Nevertheless, children with RBD affecting chest wall development (Table 2), could be at higher risk of acute respiratory insufficiency even though affected by a mild form of the disease.

**Table 2:** Some RBD associated with chest wall abnormalities

- Osteogenesis Imperfecta type III (see OMIM for various forms)	
- Vitamin D-dependent rickets type 2 (OMIM 277440; 600,785)	
- Hypophosphatasia Infantile form (OMIM 241500)	
- Cleidocranial dysplasia (OMIM 119600)	
- Osteopetrosis (see OMIM for various forms)	
- Ellis-van Creveld (OMIM 225500)	
- Jeune syndrome (OMIM 208500)	
- Diastrophic dysplasia (OMIM 222600)	
- Marfan syndrome (OMIM 154700)	
- Neurofibromatosis type 1 (OMIM 162200)	
- Mucopolysaccharidosis (see OMIM for various forms)	
- Achondrogenesis (OMIM 200600)	
- Poland syndrome (OMIM 173800)	
- Klippel-Feil syndrome (see OMIM for various forms)	
- Spondylocostal dysostosis (see OMIM for various forms)	
- Thanatophoric dysplasia (OMIM 187600;187,601)	
- Barnes symdrome (OMIM 187760)	
- Short-rib polydactyly dysplasia (see OMIM for various forms)	
- Congenital scoliosis (OMIM 122600)	

*OMIM* Online Mendelian Inheritance in Man, OMIM®

Chest wall malformations, including excessively stiff and narrow chest, can lead to a decreased functional residual capacity, exposing patients to severe respiratory insufficiency [46–50]. These patients could, therefore, be susceptible to more severe complications related to SARS-CoV-2 infection than healthy subjects. For patients with chest wall malformations it is therefore recommended to seek for medical advice since the onset of fever and to access to hospital in case of persistent, severe cough, increased respiratory rate for age, worsening breathing difficulties, and/or oxygen saturation < 97% by pulse oximetry.

It is also important to consider that symptoms of SARS-CoV-2 infection overlap with symptoms of many other pediatric diseases, and that other pediatric diseases that have not disappeared. Access to Italian Emergency Departments has decreased dramatically, as hospitals are considered possible sites of infection for COVID-19. This is leading to late admissions of pediatric patients in advanced stages of other diseases.

All age groups are at risk infection by SARS-CoV-2, but elderly people are at the highest risk of developing a more severe infection, leading to a critical illness due to the physiological changes related to ageing, and the possible underlying health comorbidities [51]. Considering that European countries have an enormous concentration of elderly people and that over 95% of all deaths caused by COVID-19 occurred in individuals older than 60 years, following all recommendations to avoid infection is crucial [1]. Furthermore, smoking represents an important factor for COVID-19 disease severe outcomes and mortality, in particular for elderly males: according to WHO, the odds of disease progression is 14 times higher among people with a history of smoking compared to those without [52].

### Psychological aspects

Despite their physical limitations and motor impairment, patients with RBD often present normal or higher degree of social, communicative and resilience skills. For example, people affected with OI often achieve high educational and employment levels [53–55]. The Quality of Life of people with OI, from childhood to adulthood, show more restrictions regarding physical activities, whereas socio-cultural and mental QoL seem to be less affected [56–58].

A high degree of resilience and a low incidence of depression are reported. Since childhood, they have learnt that their life will be marked by a long series of challenges and ups and downs [54, 59]. From that, a strong impulse to start over again after fractures or surgery, and to resume the goals previously achieved to try to overcome them emerges [53].

Behind their accomplishments and their remarkable abilities, however, an effective management of anxiety, fear of pain and risk of fractures is required. The need to feel safe in social contexts and the concern for a harm free physical contact with other people are persistent [60, 61]. Physical integrity is a source of continuous subliminal continuous stress [53].

During the recurring and extended hospitalizations for fractures and related surgeries up to home recoveries, families are involved together with healthcare professionals in the care and cure of their relatives with OI up to adult life, if total autonomy cannot be reached [58, 60, 61]. Children and adults with OI frequently don’t rely on those who do not know their bones brittleness and their functional limitations [62].

In the case of COVID-19 patients affected with RBD:

- As long as it is possible, using appropriate PPE, the presence of a family member should be fostered in order to: instruct health care staff to correctly and safely handle the patient’s body; inform on past and present difficulties and dysfunctions; help the patient with their daily routine [53–55, 62].
- At any age, patients with RBD need to be adequately informed of their conditions. Every medical procedure has to be adequately explained properly. These patients are often hugely self-aware and well-informed on their RBD. Even a child can be a major ally with the health care team if actively involved and sufficiently informed of his/her treatment [60].
- A hearing impairment should be considered when communicating with the patient to avoid the feeling of isolation and misunderstandings [53, 58].
- RBD patients can give relevant suggestions to health care staff to handle their body and about their pathology characteristics and limitations. Never underestimate an active listener [55, 58].
- Short stature is common in adult OI patients, but it should be always be avoided to treat them like children. On the other hand, it should be remembered that children with OI are usually more self-aware than their peers [58].
- Due to the intense stress for the risk of fractures and death, often already experienced in previous hospitalizations, patients are at a high risk of post-traumatic stress disorder (PTSD). Psychological support for the patients and their families might be advisable [55, 60–63].

### The role of patients’ associations

Patient Associations have always been a point of reference for their communities. The comparison and dialogue among associates are the main activity of the Associations and today this occurs more and more frequently through ways of communication such as video conferences or social networks. Face-to-face national or international meetings and conferences are important too, at the presence of clinicians, specialists and representatives of the Institutions.

These sharing tools or methods allow the Associations to create a complete and as comprehensive as possible perspective, relating to the needs of the patient himself, offering better data collection.

Currently many Associations have developed processes and methodologies to create an effective “empowerment” that allows its members to be fully prepared and involved in sectors such as research, biobanking and clinical studies and to participate in trials.

Collaboration among Associations is fundamental for their growth, as well as a direct comparison during congresses or video conferences among patients and specialists from Universities or research and clinical centers. Nowadays, the Associations are responsible for gathering the needs of the rare diseases patients in order to be able to:

- deal with and discuss at the technical tables with the Institutions, in order to implement decisions “patients-oriented” decisions (2) to compare them with what has been done in the Reference Centers.

The Associations also represent a point of reference in times of emergency, providing broad spectrum support to the members in need. Cases in which patients’ requests to the COVID-19 Helpline cannot be resolved from a medical / scientific point of view are referred to the Patient Associations. Thus, they can provide prompt responses to people who have expressed difficulties and place them in an extended network context that also includes legal, social and psychological support.

For this purpose, the COVID-19 Helpline is connected to other local networks, such as Uniamo’s SAIO Helpline (Listening, Information and Guidance Service) and an As.It.O.I. active phone number, with telephone, message service and voicemail available. In the COVID-19 emergency, the problems to be addressed can be different and can be summarized in:

(i) care and intervention aspects directly related to rare diseases
(ii) psychological aspects that are generated or amplified by the emergency itself
(iii) social and legal aspects that impact on the working and relationship life of the concerned person.

The Associations provide real-time feedback on the treatments requested and obtained treatments, thus representing a litmus test for the institutions that must take care of the patients with a rare disease.

## Conclusions

The present position statement highlights the fundamental role of remote high quality of care for RBD during the COVID-19 outbreak. The Italian RBD expert group recommends to extend to other countries the successful experience of the COVID-19 Helpline for RBD. Rare diseases other than RBD could benefit from similar services and harmonization of gold-standard practices for remote care. Further information on Italian and European COVID-19 Initiatives can be found in the ERN BOND website ernbond.eu.

## Data Availability

Not applicable.

## Abbreviations

A&E: Accident and Emergency department
ARF: Acute Respiratory Failure
As.It.O.I.: Italian Osteogenesis Imperfecta Association
ePAGS: European Patient Advocacy Groups
ERN BOND: European Reference Network on Rare Bone Diseases
ETI: Endotracheal intubation
FiO_2_: Fraction of Inspired Oxygen
HFNC: High-Flow Nasal Cannula
IMV: Invasive Mechanical Ventilation
NIV: Non-Invasive Ventilation
OI: Osteogenesis Imperfecta
PPE: Personal Protective Equipment
PTSD: Post-traumatic stress disorder
RBD: Rare bone diseases
Vt: Low Tidal Volume
WHO: World Health Organization
